# Roles of MPBQ-MT in Promoting α/γ-Tocopherol Production and Photosynthesis under High Light in Lettuce

**DOI:** 10.1371/journal.pone.0148490

**Published:** 2016-02-11

**Authors:** Yueli Tang, Xueqing Fu, Qian Shen, Kexuan Tang

**Affiliations:** Fudan-SJTU-Nottingham Plant Biotechnology R&D Center, School of Agriculture and Biology, Shanghai Jiao Tong University, Shanghai, China; Institute of Genetics and Developmental Biology, Chinese Academy of Sciences, CHINA

## Abstract

2-methyl-6-phytyl-1, 4-benzoquinol methyltransferase (MPBQ-MT) is a vital enzyme catalyzing a key methylation step in both α/γ-tocopherol and plastoquinone biosynthetic pathway. In this study, the gene encoding MPBQ-MT was isolated from lettuce (*Lactuca sativa*) by rapid amplification of cDNA ends (RACE), named *LsMT*. Overexpression of *LsMT* in lettuce brought about a significant increase of α- and γ-tocopherol contents with a reduction of phylloquinone (vitamin K1) content, suggesting a competition for a common substrate phytyl diphosphate (PDP) between the two biosynthetic pathways. Besides, overexpression of *LsMT* significantly increased plastoquinone (PQ) level. The increase of tocopherol and plastoquinone levels by *LsMT* overexpression conduced to the improvement of plants’ tolerance and photosynthesis under high light stress, by directing excessive light energy toward photosynthetic production rather than toward generation of more photooxidative damage. These findings suggest that the role and function of *MPBQ-MT* can be further explored for enhancing vitamin E value, strengthening photosynthesis and phototolerance under high light in plants.

## Introduction

**Fig 1:**
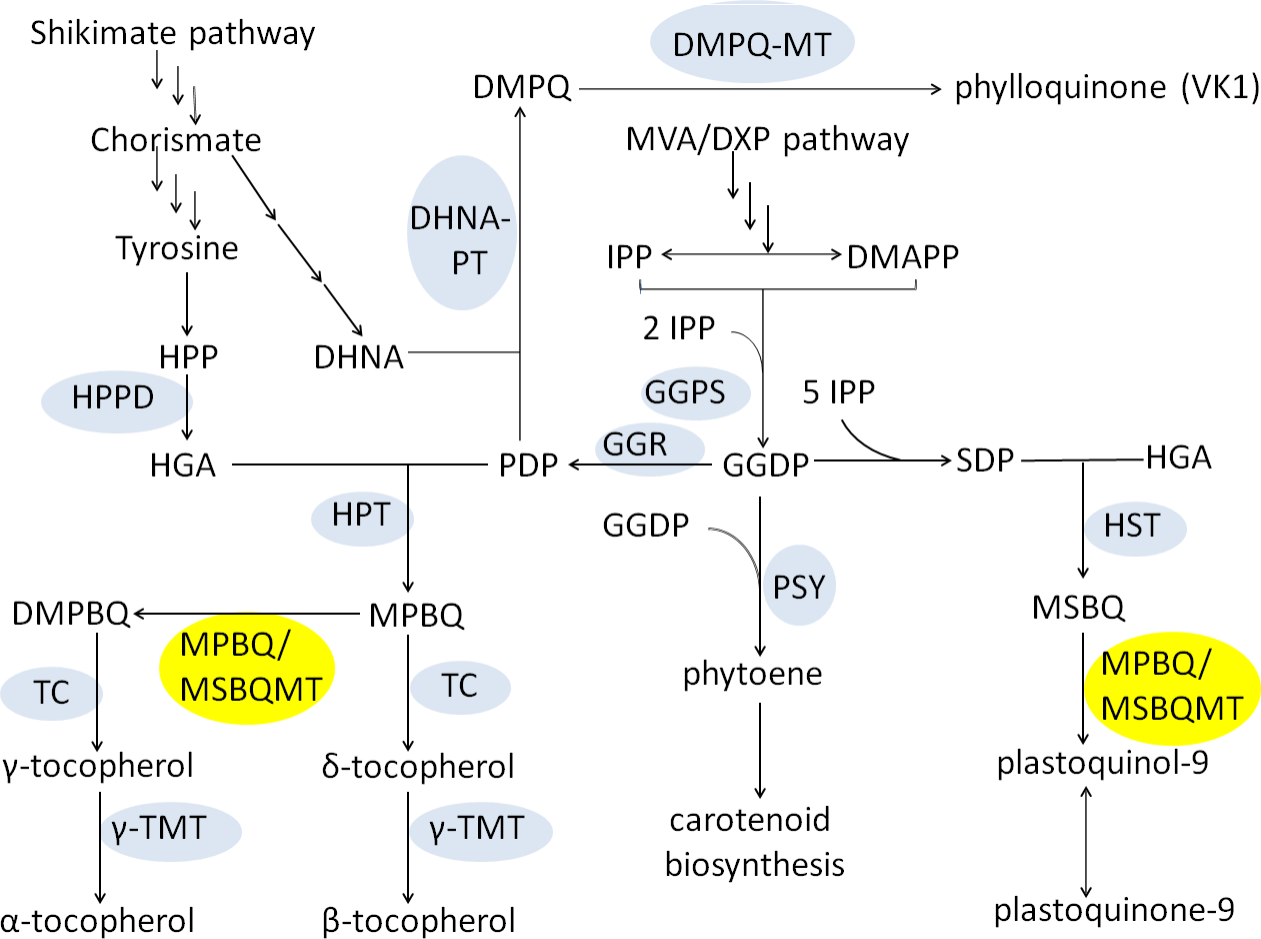
Biosynthetic pathways of vitamin E, vitamin K1, carotenoid and plastoquinone. Substrate abbreviations: DHNA, 1,4-dihydroxy-2-naphtoate; DMPBQ, 2,3-dimethyl-5-phytyl benzoquinol; DMPQ, demethylphylloquinone; DMAPP, dimethylallyl diphosphate; GGDP, geranylgeranyl diphosphate; HGA, homogentisate; HPP, ρ-hydroxyphenylpyruvate; IPP, isopentenyl diphosphate; MPBQ, 2-methyl-6-phytyl-1,4-benzoquinol; MSBQ, 2-methyl-6-solanesyl-1,4-benzoquinol; PDP, phytyl diphosphate; SDP, solanesyl diphosphate. Enzyme abbreviations: DHNA-PT, DHNA phytyl transferase; DMPQ-MT, DMPQ methyltransferase; GGPS, geranylgeranyl diphosphate synthase; GGR, geranylgeranyl reductase; HPPD, HPP dioxygenase; HPT, homogentisate phytyl transferase; HST, homogentisate solanesyl transferase; MPBQ/MSBQ-MT, MPBQ/MSBQ methyltransferase; PSY, phytoene synthase; TC, tocopherol cyclase; γ-TMT, γ-tocopherol methyltransferase.

*MPBQ-MT* is an important gene involved in both tocopherol (vitamin E) and plastoquinone biosynthesis (Fig 1), which was first isolated from and characterized in *Arabidopsis thaliana* by screening of mutants deficient in α-/γ-tocopherol and plastoquinone [1, 2]. In tocopherol biosynthesis, homogentisate (HGA) from the Shikimate pathway is condensed with phytyl diphosphate (PDP) by catalyzation of homogentisate phytyl transferase (HPT) to form 2-methyl-6-phytyl-1, 4-benzoquinol (MPBQ) [3, 4]. Then the biosynthetic pathway diverges: 1) MPBQ is converted to 2, 3-dimethyl-5-phytyl benzoquinol (DMPBQ) by MPBQ methyltransferase (MPBQ-MT) and then cyclized to form γ-tocopherol and further methylated into α-tocopherol by the catalyzation of tocopherol cyclase (TC) and γ-tocopherol methyltransferase (γ-TMT) sequentially. 2) MPBQ is straight cyclized into δ-tocopherol and further methylated into β-tocopherol by TC and γ-TMT sequentially. MPBQ-MT functions at the keypoint of the tocopherol metabolic flux, which directs the flux toward γ- and α-tocopherol biosynthesis. On the other hand, condensation of HGA with solanesyl diphosphate (SDP) forms 2-methyl-6-solanesyl-1, 4-benzoquinol (MSBQ), which is then methylated into plastoquinol (PQH2, the reduced form of plastoquinone) by MPBQ-MT (Fig 1) [5, 6].

Both plastoquinone and tocopherol play important physiological roles in plants. Plastoquinone (PQ) is an essential electron carrier shuttling between photosystem II (PS II) and Cytb6f in the electron transport chain (ETC) of photosynthesis in the chloroplast. It’s believed that the redox state of plastoquinol pool regulates phosphorylation of light-harvesting complexes (LHC) via a Cytb6f kinase [7–9], chloroplastic gene expression of proteins of PS II and PS I complexes [10–13], nuclear encoded gene expression for ascorbic peroxidase (APX1/2) [14–17] and superoxide dismutase (SOD) [18] that function in scavenging reactive oxygen species (ROS) under stress. Moreover, plastoquinol itself could act as a quencher of singlet oxygen generated via triplet chlorophyll in the reaction centre of photosystems during high light stress [19] and its antioxidant activity has been suggested in several studies [20–22]. The function of tocopherols has been researched for decades. Its best known function lies in its powerful antioxidant activity responsible for scavenging ROS such as singlet oxygen (O2*), superoxide (O2⁻) and lipid hydroxyl radicals (-OH) that would otherwise cause damage and degradation of membrane lipids, proteins in photosystems, and other components in the cell [23–26].

Tocopherols can be found widely in diverse tissues of plants like leaves, seeds, fruits, stems and so on. In general, α- and γ-tocopherols are the main form of tocopherols in photosynthetic tissues, while δ- and β-tocopherols merely account for a very small proportion [27, 28]. Tocotrienols appear to be less widespread, which are present predominantly in monocot seeds [28, 29] and have also been identified in leaves of some monocots recently [30]. The α-tocopherol has the highest vitamin E activity due to its preferential retention by the tocopherol transfer protein in animals [23, 31]. Therefore, α-tocopherol is the most regarded and studied form of all tocopherols for the sake of improving nutrient value of human diets. Many genetic manipulations of related genes have been conducted in the past decades for enhancing vitamin E value in plants, including overexpression of single or multiple genes encoding rate-limiting enzymes in tocopherol biosynthesis, introduction of transcription factor enhancing expression of these genes, and RNAi/antisense knockout of genes involved in another competing metabolic pathway. With these strategies, total vitamin E content could be increased, or the vitamin E composition could be alterd in favor of α-tocopherol [5, 6]. But these researches paid little attention to the influence of vitamin E-elevating strategy on other possibly important metabolites or nutrients.

In this study, we created transgenic lettuce overexpressing *LsMT*, which had higher α/γ-tocopherol contents but lower vitamin K1 content than non-transgenic ones. This suggests that strengthening the expression of genes functioning upstream in metabolic fluxes to increase the substrate synthesis is probably an effective way for both enhancing target nutrient’s production and maintaining other nutrients’ contents as well. Moreover, we explored the possible mechanism for the transformant’s stronger tolerance and increased photosynthetic product under high light stress, and proposed a valid model as an interpretation of this phenomenon. Our study offers a good example for increasing target nutrient value of plants with increased photosynthesis.

## Results and Discussion

### Isolation and characterization of *MPBQ-MT* gene from lettuce

The coding sequence of *MPBQ-MT* from lettuce (*LsMT*) was isolated by rapid amplification of cDNA ends (RACE) and its full length was obtained by genomic DNA amplification. Its coding region is 1014bp long, divided into 3 exons by 2 introns (S1 Fig). It encodes a polypeptide consisting of 337 amino acids (Accession No. in Genbank: ACP43457). The alignment of protein sequence showed an identity of 76–86% in sequence between LsMT and MPBQ-MTs from other plant species. The mere exception is *Chlamydomonas reinhardtii*, whose MPBQ-MT is only 66% identical to LsMT in sequence (S2 Fig). Meanwhile, LsMT was predicted to have an N-terminal chloroplast transit peptide (cTP) of 52 residues. Close similarity in amino acid sequence and the same location in chloroplasts as previously reported MPBQ-MTs suggested that *LsMT* isolated from lettuce is the homologue to those from other species encoding MPBQ-MT.

### Overexpression of LsMT causes an increase of α- and γ-tocopherol levels with decreased phylloquinone (VK1) level in lettuce leaves

**Fig 2:**
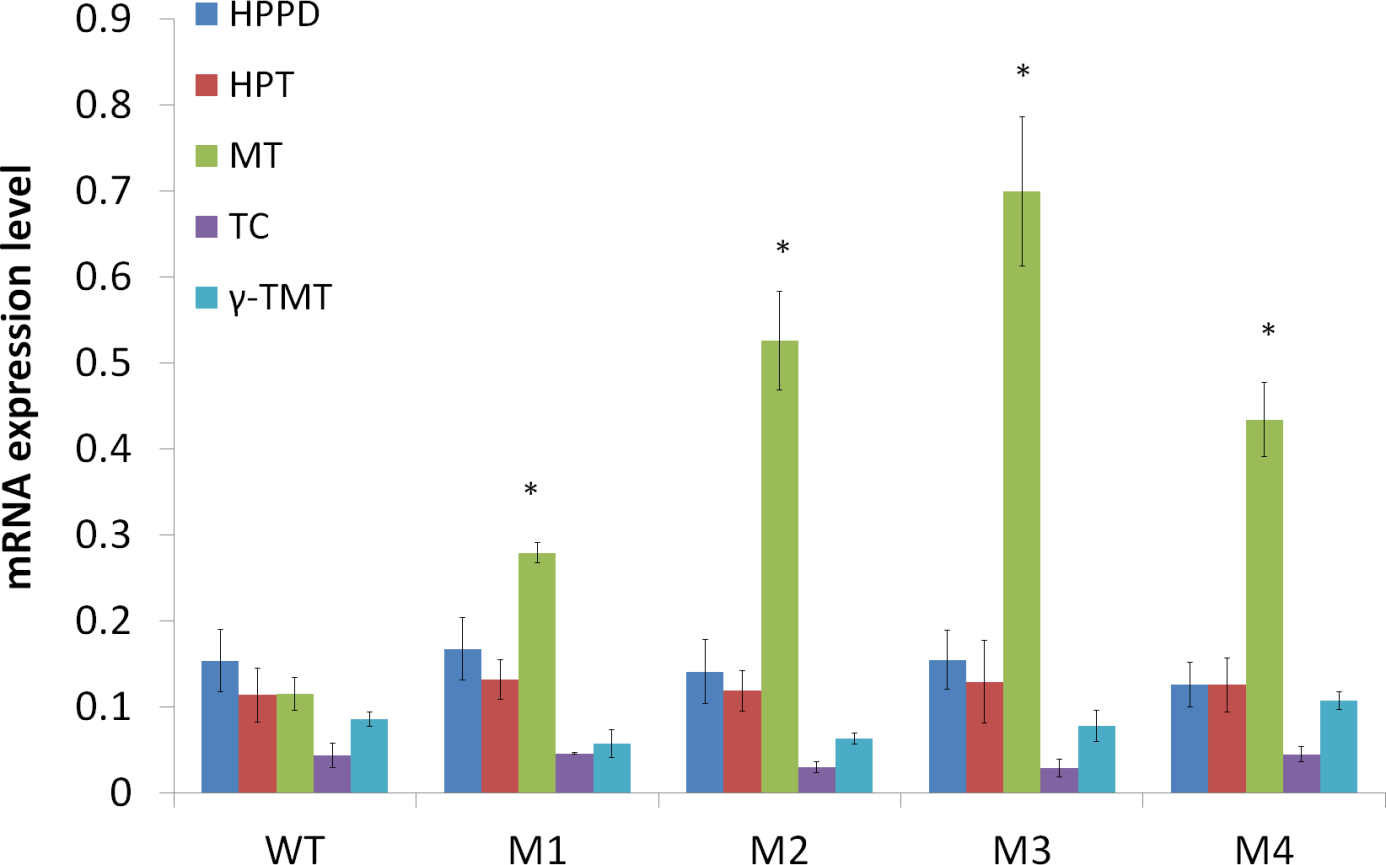
Expression levels of genes encoding HPPD, HPT, MPBQ-MT, TC and γ-TMT involved in tocopherol biosynthesis in wild type (WT) and transgenic lines (M1-M4), measured by quantitative real-time PCR. *Ubiquitin* was used as a control for normalization. mRNA expression levels (y-axis) of the five genes were measured with 2^-ΔCt^. Data is the mean value ± SD of four biological replicates from T1 progenies. Asterisk indicated a significant difference compared to the value of WT (Student’s *t*-test, P<0.05).

**Fig 3:**
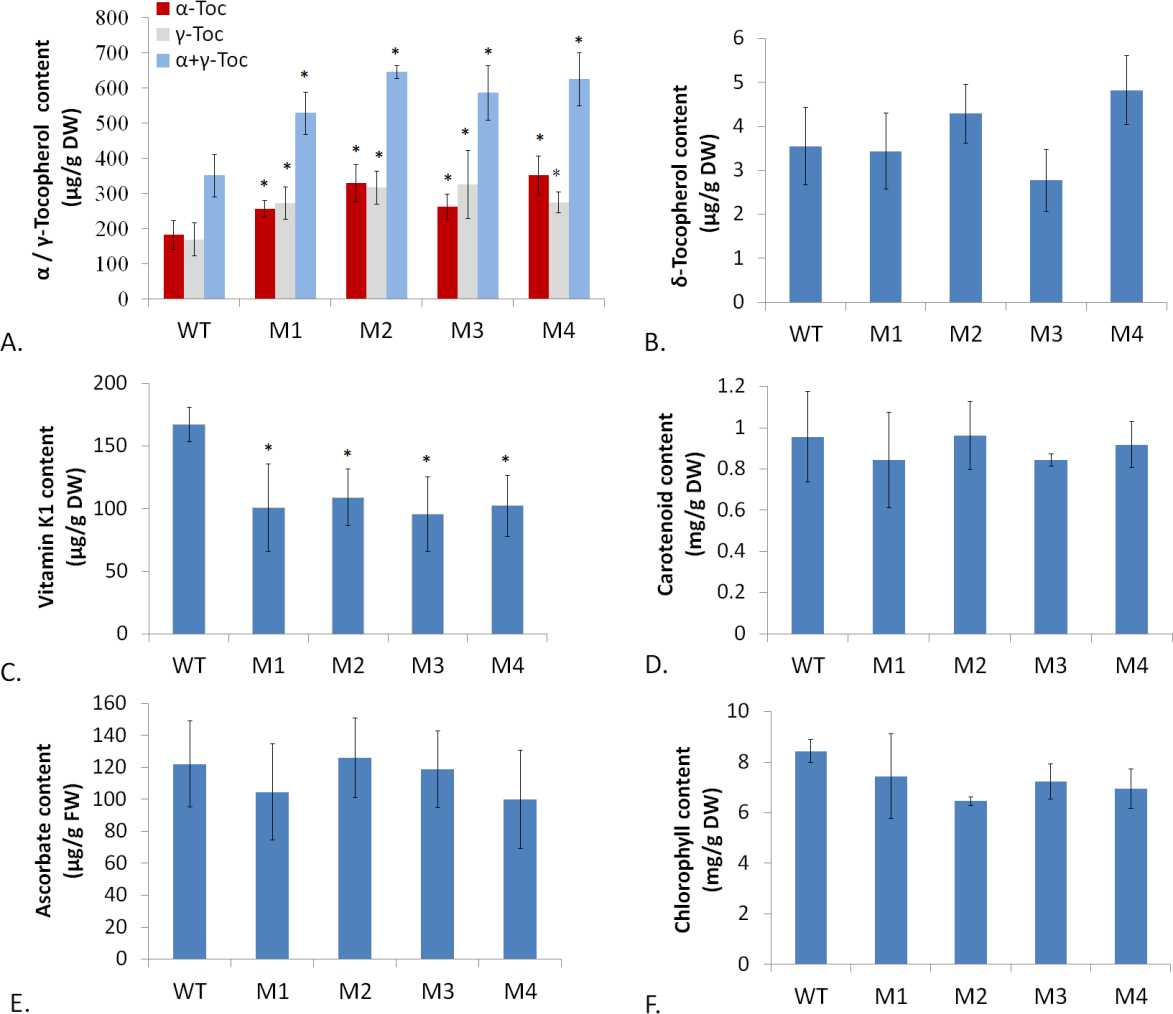
**Analyses of (A) α-, γ-, (α+γ)-tocopherol content, (B) δ-tocopherol content, (C) phylloquinone content, (D) carotenoid content, (E) ascorbate content and (F) chlorophyll content in wild type (WT) and transgenic lines (M1-M4).** Data is the mean value ± SD of four biological replicates from T1 progenies. Asterisk indicated a significant difference compared to the value of WT (Student’s *t*-test, P<0.05).

To examine the function of *LsMT* in enhancing tocopherol biosynthesis, we overexpressed this gene in lettuce by means of Agrobacterium-mediated transformation with pCAMBIA 2300 vector. Four independent transgenic lines (M1-M4) were selected out in kanamycin-containing medium and further confirmed by genomic PCR detection (S3 Fig). Then they were kept for further analysis. Quantitative PCR (qPCR) result showed the transcription level of *LsMT* in transgenic plants increased by 1.5–5 folds, compared to that in wild type plants (Fig 2). We also analyzed the expression levels of *HPPD*, *HPT*, *TC*, *γ-TMT*, to see if the upregulation of *LsMT* expression in transgenic plants has any impact on the expression of these up- and downstream genes in the tocopherol biosynthetic pathway (Fig 2). The result showed there is no significant difference in the expression levels of *HPPD*, *HPT*, *TC* and *γ-TMT* between wild type and transgenic plants, indicating that overexpression of *LsMT* does not impact on the expression of other genes up- and downstream in the tocopherol biosynthetic pathway. Then tocopherols’ content in wild type and transgenic plants was analyzed by HPLC. The result indicated that the transformants contained a 60%-93% higher level of γ-tocopherol and 41%-93% higher level of α-tocopherol than the wild type. And the total (α+γ)-tocopherol content was raised by 50–84% due to *LsMT* overexpression (Fig 3A). These results demonstrated that *LsMT* overexpression could significantly enhance α- and γ-tocopherol biosynthesis in lettuce. However, δ-tocopherol synthesis in the other metabolic branch was not downregulated by α- and γ-tocopherol enhancement (Fig 3B), although the two different pathways shared a common upstream substrate MPBQ in somewhat competitive relations that could be seen in Fig 1. This phenomemon appeared not to be consistent with an earlier report, in which overexpressing *MPBQ-MT* from *Arabidopsis thaliana* in soybean led to reduced δ-/β-tocopherols with a proportionate increase in γ-/α-tocopherol levels in seeds [1]. Soybean seeds are rich in all kinds of tocopherols, and in this study, the amount of increase in (α+γ)-tocopherol content was nearly consistent with that of decrease in (δ+β)-tocopherol content. But in lettuce leaves, δ- and β-tocopherols only account for a quite marginal portion of total tocopherols, which are easily overlooked. So the visible α- and γ-tocopherol enhancement by *LsMT*-overexpression can’t be owed to marginal sacrifice of low-leveled δ- and β-tocopherol biosynthesis. And this result ultimately determined the rise of total tocopherol levels in transgenic lettuce leaves. Hence we speculated there should be some other metabolic pathways influenced by tocopherol increase, which possibly need the same substrate upstream as tocopherol biosynthetic pathway.

To detect whether tocopherol enhancement affected other vitamins’ levels, we selected carotenoid (provitamin A), ascorbate (vitamin C), and phylloquinone (vitamin K1) as target analytes in our study. Our data showed both carotenoid and ascorbate contents were not affected by tocopherol enhancement (Fig 3D and 3E), while phylloquinone level was downregulated by about 30%-40% in transgenic plants, relative to wild type (Fig 3C). Both phylloquinone and tocopherol biosyntheses need a common substrate PDP upstream in metabolic fluxes and they have to compete for this substrate (Fig 1). This may explain why the upregulation of tocopherol level in transgenic plants brought about a visible downregulation of phylloquinone level. Meanwhile, the content of chlorophyll, an essential participant in photosynthetic process, was measured, whose biosynthesis also needs PDP as a substrate. The result showed there was no significant difference statistically between chlorophyll levels in wild type (WT) and in transgenic plants (TR) (Fig 3F). This result suggested that no significant competition for substrate as PDP occurred between chlorophyll and tocopherol biosyntheses, at least not to an extent that evidently affected chlorophyll biosynthesis by tocopherol enhancement.

The concomitant decrease of phylloquinone with tocopherol increase in transgenic plants suggests that enhancing the substrate production required for multiple biosyntheses is probably an effective solution to increase target product synthesis without sacrificing or weakening other metabolic fluxes. For the goal of ample substrate supply, fortifying the expression of genes which function upstream in the substrate synthesis pathway is a helpful method. An earlier report has demonstrated that overexpressing the gene of 1-deoxy-D-xylulose-5-phosphate reductoisomerase (DXR) in tobacco contributed to elevation of various isoprenoid levels like chlorophyll, phytol, carotenoid, solanesol and so on [32]. Following such examples, we could try to elevate expression levels of genes encoding DXR or geranylgeranyl reductase (GGR) involved in PDP biosynthesis upstream to supply ample substrate for both tocopherol and phylloquinone production. Moreover, enhancing the level of chorismate, the precursor for synthesizing both HGA and 1, 4-dihydroxy-2-naphtoate (DHNA) in tocopherol and phylloquinone pathways respectively, may also have an impact, though possibly to a slight extent, since chorismate lies far upstream in Shikimate pathway (Fig 1). Anyhow, all these ideas deserve further practice and exploration in our efforts for breeding and cultivation of high nutrient-valued vegetables.

### Overexpression of *LsMT* causes an elevation of plastoquinone level in lettuce

**Fig 4:**
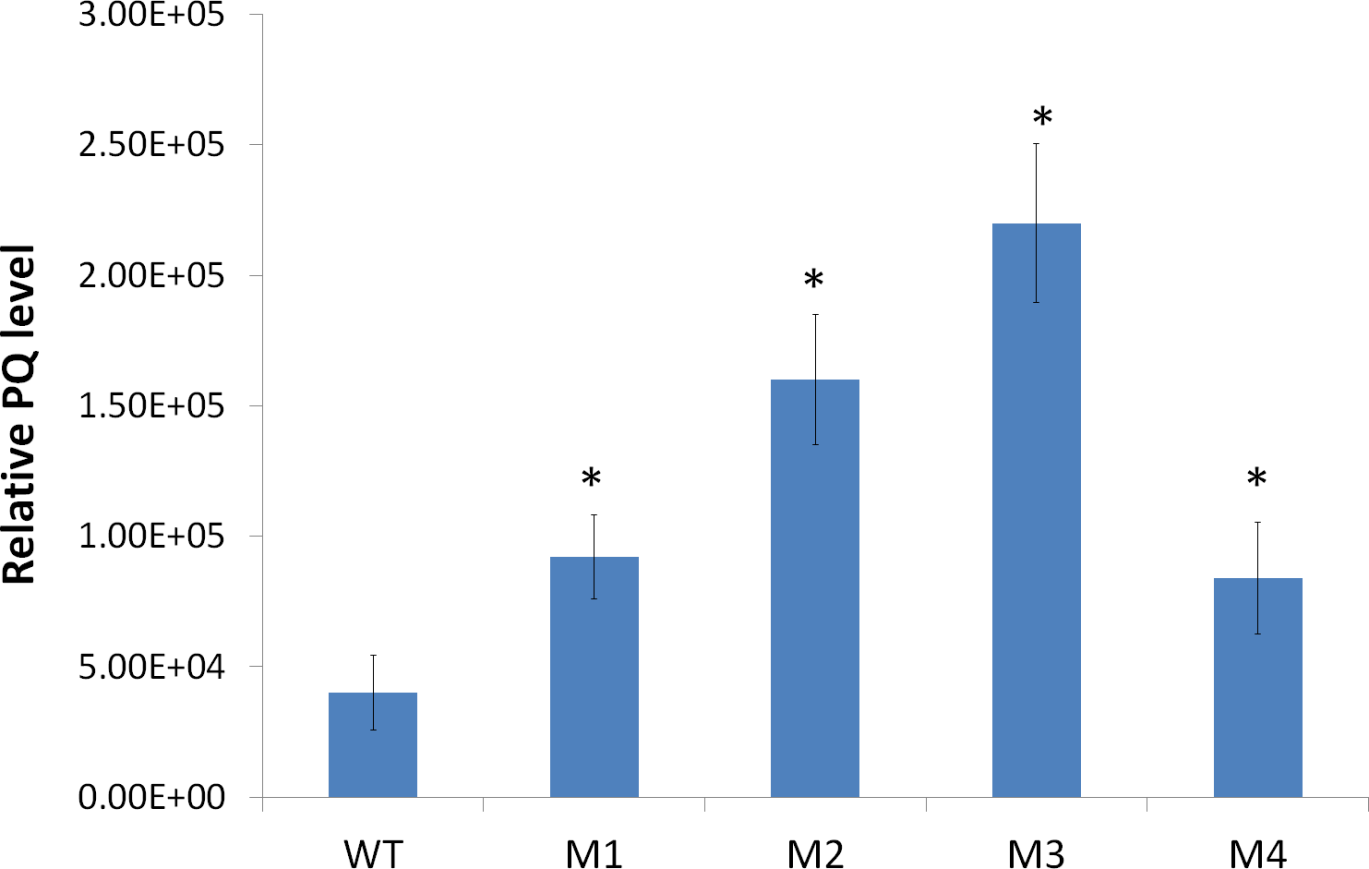
Relative plastoquinone level in wild type (WT) and transgenic lines (M1-M4). Data is the mean value ± SD of four biological replicates from T1 progenies. Asterisk indicated a significant difference compared to the value of WT (Student’s *t*-test, P<0.05).

A previous study showed null-*MPBQ-MT* mutants of *Arabidopsis* were deficient in both plastoquinone and α-/γ-tocopherols, which demonstrated a vital part of MPBQ-MT in maintaining plastoquinone and tocopherol production [2]. However, so far no study has been conducted to examine the effect of *MPBQ-MT* overexpression on PQ pool content. To detect the impact of overexpression of *LsMT* in lettuce on PQ pool level, we extracted it from leaves of transgenic and wild type plants, and analyzed its relative level by Ultra-Performance Liquid Chromatography & Triple Quadrupole Mass Spectrometer (UPLC-3Q-MS). Because plastoquinol was quite unstable and easily oxidized into plastoquinone during extraction, we finally analyzed plastoquinone level in different samples. Since we had no plastoquinone or plastoquinol standards, we selected specific parent ion at *m/z* 749.6 and daughter ion at *m/z* 151.1 peculiar to PQ molecule, and performed multiple-reaction monitoring (MRM) scan for plastoquinone detection in positive ion mode, referring to [33, 34]. By contrasting the intensity of signal peaks that reflects PQ level in different samples, we could do relative quantification of PQ level. As shown in Fig 4, the intensity of PQ signal peaks from transgenic samples was 2–4 fold higher than that of wild type, reflecting a proportionate elevation of PQ level in transgenic plants. Such a result suggested that overexpression of *LsMT* could significantly elevate PQ biosynthesis. This implies that MPBQ-MT is a key enzyme as decisive for PQ biosynthesis as for α-/γ-tocopherol biosynthesis.

### *LsMT*-overexpressing plants showed stronger resistance to high light stress with increased photosynthesis product than wild type

**Fig 5:**
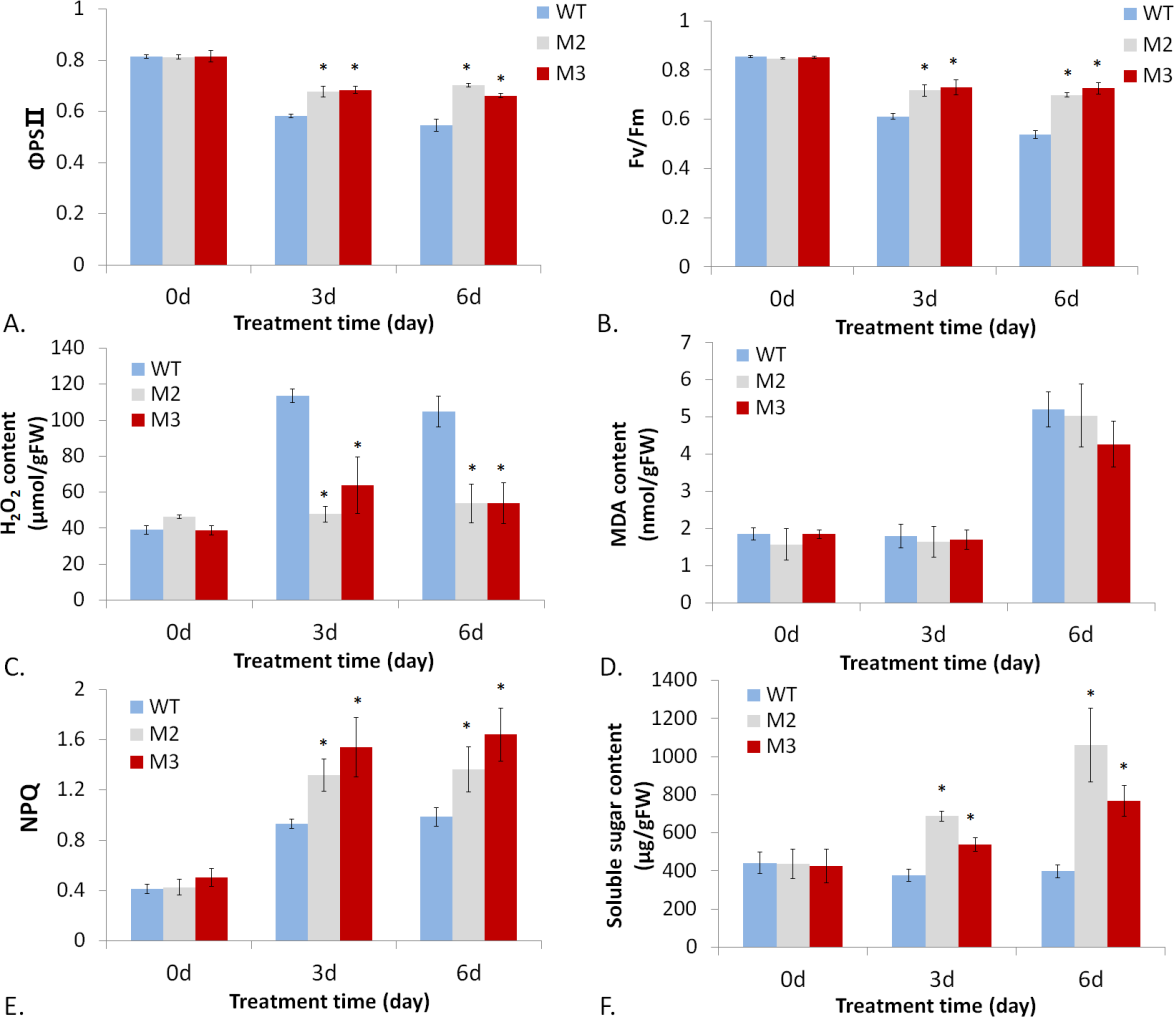
Measurements of chlorophyll fluorescence, photooxidation products, NPQ level and soluble sugar content in wild type (WT) and transgenic plants’ leaves under high light condition (photon flux density: 1000μmol/m^2^/s, 16h light/8h dark) for 6 days. The measurements were taken after 0, 3, 6 days of the treatment. (A) the actual quantum yield of PSⅡ-mediated electron transport, (B) the maximum quantum yield of PSⅡ photochemistry, (C) H_2_O_2_ content, (D) malondialdehyde (MDA) content, (E) NPQ level and (F) soluble sugar content. Data is the mean value ± SD of four biological replicates from T1 progenies. Asterisk indicated a significant difference compared to the value of WT (Student’s *t*-test, P<0.05).

Plants absorb light energy for photosynthesis. When absorbed light energy exceeds the need of photosynthesis for carbon fixation, such excessive energy would be managed through several mechanisms: 1) transfer to O_2_ through triplet state chlorophyll (Chl) from singlet state Chl, yielding singlet oxygen (O_2_*); 2) thermal dissipation (also termed non-photochemical quenching, NPQ) involving pH gradient accumulation across the thylakoid membrane and zeaxanthin; 3) H_2_O-H_2_O cycle as alternative sinks for excessive photons and electrons involving redox switch of ascorbate; 4) cyclic electron transport chain (ETC) recycling excessive electrons back to PQ from NADPH or ferredoxin (Fd) to relieve excessive electron pressure and prevent overreduction or inactivation of PS I; 5) photorespiration and malate valve as an outlet of excessive electron energy (S5 Fig) [35–40]. And those ROS generated in above-mentioned energy-dissipating pathways are subsequently scavenged or quenched by antioxidants like carotenoid, ascorbate and tocopherols. Besides, PQ has been reported to act as an antioxidant and contribute to plant tolerance to excess light energy and photodamage [41]. To test whether transgenic lettuce plants (TR) containing more tocopherols and larger PQ pools have better tolerance to photodamage under high light conditions, we selected out two transgenic lines (M2 and M3) showing the highest mRNA expression level, and subjected them to high light treatment together with wild type (WT). The two lines and wild type control were grown under high light conditions (photon flux density: 1000μmol/m^2^/s, 16h light/8h dark) for 6 days. We surveyed the maximum quantum yield of PS II (Fv/Fm), the actual efficiency of PS II photochemistry (ΦPS II), thermal dissipation (also termed non-photochemical quenching, NPQ) efficiency, contents of H_2_O_2_, malondialdehyde (MDA), and soluble sugars after 0, 3, 6 days of the high light duration. As shown in Fig 5A and 5B, high light treatment brought about a modest and continuous decline of Fv/Fm and ΦPS II values in both WT and TR. But the decline of Fv/Fm and ΦPS II values in WT is more drastic than that in TR. The relatively more drastic drop of Fv/Fm and ΦPS II values in WT indicated that PS II in WT suffered a more severe photoinhibition under high light than that in TR. This means the photosystem of TR could work better or more efficiently under high light stress than that of WT.

To test whether TR had stronger resistance to photooxidative damage than WT, we detected H_2_O_2_ and MDA levels in cells of WT and TR, the products of photooxidation generated under high light stress. The result indicated that high light treatment caused a significant increase in H_2_O_2_ level in WT, but only a minor increase in TR. H_2_O_2_ level rose sharply by 2 fold in WT, but rose modestly by 20%-30% in TR under high light (Fig 5C). This result clearly demonstrated higher photooxidation resistance, or exactly speaking, stronger ROS removing capacity of TR under high light stress. MDA, the product of lipid peroxidation induced by ROS like H_2_O_2_, O_2_*, O_2_^⁻^, -OH, didn’t show visible increase after 3 days’ treatment, but increased significantly after treatment for 6 days in both WT and TR (Fig 5D). But there was no significant difference in MDA levels statistically between WT and TR during high light treatment. H_2_O_2_ is mainly generated from superoxide (O_2_⁻) via H_2_O-H_2_O cycle under SOD catalyzation. Under high light stress, O_2_⁻ is produced within thylakoid membranes. Besides being scavenged by ascorbate and SOD, it can also be scavenged by α-tocopherol and plastoquinol [42, 43]. More α-tocopherol and plastoquinol supply could scavenge more O_2_⁻ in TR, thus reducing H_2_O_2_ generation. This may be able to account for lower H_2_O_2_ level in TR than in WT.

In several studies, MDA levels do not correlate with the intensity of the applied stress [44]. And MDA is not the mere product of lipid peroxidation. Other oxylipins, such as hydroxy octadecatrienoic acids (HOTEs), are also main products of lipid peroxidation, which will give more precise and exclusive information on photo-oxidation related process under high light stress [45, 46]. Therefore, mere MDA levels may not be sufficient to comprehensively reflect the exact extent of lipid peroxidation in plants. Other oxylipins, such as HOTEs, also need to be considered in the future research.

Then NPQ values of chlorophyll fluorescence were measured in WT and TR leaves. As shown in Fig 5E, thermal dissipation (measured by NPQ value) was significantly strengthened under high light condition in both WT and TR leaves. But TR leaves had a significantly higher NPQ level, about 40% and 60% higher in M2 and M3 respectively, than WT under high light stress. This result indicated higher thermal dissipation efficiency of TR under high light, compared to WT. The stronger ROS removing capacity and higher thermal dissipation efficiency of TR suggested that excessive light energy can be better released via ROS-scavenging (by more tocopherol/plastoquinol) and thermal dissipation (higher NPQ) pathways in TR. So the photosystem (PS) in TR suffered lesser photodamage or photoinhibition by excess light energy, and had relatively higher Fv/Fm and ΦPSⅡvalue than WT. Our results were consistent with the previous report, which demonstrated that transgenic *Arabidopsis* plants accumulating more PQ suffered lesser photooxidative damage with higher PS Ⅱ photochemical efficiency than WT under high light stress [41]. Finally, we detected soluble sugar content in WT and TR during high light treatment (Fig 5F). Soluble sugar level of WT showed a minor drop after 3 days and recovered back partially after 6 days during the treatment. On the contrary, soluble sugar level in TR rose gradually and visibly during the treatment, increasing by 1.5 fold at the most after treatment for 6 days. This result indicated that TR had significantly increased photosynthetic production during high light treatment, compared to WT. Concluded from the experimental results, TR suffered lesser photooxidative damage and photoinhibition under high light stress, with higher photosynthetic product level than WT. Such phenomena indicated that TR, with higher levels of antioxidants as tocopherols and larger PQ pool than WT, had enhanced capacity to cope with photodamage in a way of thermal dissipation and removing excessive ROS induced by excessive photon energy. And such harmful energy was ultimately directed toward photosynthetic production over the long haul. This is a course that could be called” turning the harmful into the useful”. But WT, inefficient in managing excessive energy, had to suffer photodamage more severely than TR.

**Fig 6:**
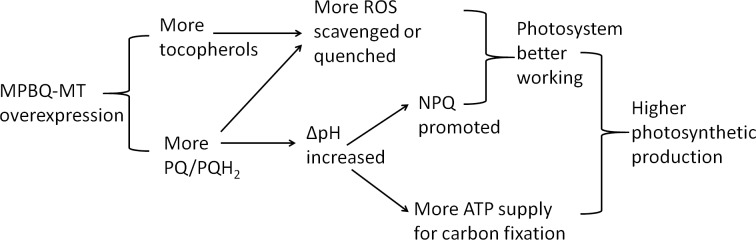
Illustration of the mechanism by which overexpression of MPBQ-MT leads to enhanced photosynthesis and tolerance to photodamage under high light in lettuce.

According to the above experimental results, we proposed a reasonable model as an interpretation of the mechanism by which TR had enhanced photosynthesis and tolerance to photodamage under high light (Fig 6). Under an extremely high light condition, TR was more competent to scavenge the substantial ROS with higher level of antioxidants like tocopherols and PQH_2_ relative to WT. Moreover, the larger PQ pool in TR was more capable to accept and hold more electrons derived from excessive excitation pressure from the ETC. Then PQ was reduced to PQH_2_. More PQH_2_ supply could contribute more H^+^ into the lumen, thus further increasing the pH gradient (ΔpH) across the thylakoid membrane. The increase of ΔpH could further promote NPQ and boost ATP formation (S5 Fig and Fig 6). In a word, in *LsMT*-overexpressing plants there was a highly regenerating mechanism to keep a higher PQH_2_ supplementation for fulfilling the function as a singlet oxygen quencher and hydrogen shuttle in the whole photosynthetic ETC. As a result, higher levels of NPQ efficiency and tocopherol/PQH_2_ as antioxidants better protected photosystems from photooxidative damage and photoinhibition caused by excessive excitation energy. And more ATP formation ultimately provided extra energy for carbon fixation. Both healthier photosystems and more ATP supply conduced to higher photosynthetic production in TR than WT (Fig 6). In the long run, for TR, excessive photon energy that should have caused photodamage ultimately flowed into photosynthetic pathway and was stored in carbohydrates. But WT, lacking so powerful antioxidant activity and so large a PQ pool as TR, had to suffer the adverse effect of excessive light energy more severely than TR. Our experimental data provided valid support for the interpretation of the mechanism, from enhanced tocopherol and PQ levels to enhanced photosynthesis and tolerance under high light stress. However, in-depth study on detailed and complicated mechanism on the molecular level is still needed in the future.

To conclude, overexpressing *LsMT* enhanced α-/γ-tocopherol and plastoquinone biosynthesis in lettuce. Such lettuce plants had higher tocopherol value and improved resistence to photodamage and strengthened photosynthesis under high light. Our study provided a novel view for people’s efforts toward improving nutrients quality, promoting photosynthetic production and elevating resistance to environmental stress in vegetables.

## Materials and Methods

### Isolation and characterization of *LsMT* from lettuce

*L*. *savita* was grown at 25°C for day/18°C for night in a chamber (16h light/8h dark). Total RNA was extracted from leaves of three-month-old plants. Full-length cDNA of *LsMT* was cloned by means of rapid amplification of cDNA ends (RACE) according to the protocol of the SMART^TM^RACE cDNA Amplification Kit (Clontech, USA). The primers MT-GSP1 (5’-GCCTCGTTGTGGGTCCGGCCAGTAC-3’) and MT-GSP2 (5’-GGTGGATGTTGGTGGAGG CACTGGG-3’) were used for 5’- and 3’-RACE respectively, with 5’- or 3’-RACE-Ready cDNA as the template. By sequencing and alignment of 5’- and 3’-RACE products, full-length coding region of *LsMT* was obtained and amplified with primers MT-ORF1 (5'-GGATCCATGGCTTCGTCGATGCTCTAT-3') and MT-ORF2 (5'-GAGCTCTCAAATTG GTTGACCTTTTGG-3'). With genomic DNA as the template, full-length of *LsMT* gene, including exons and introns, was amplified with primers MT-ORF1 and MT-ORF2. Sequence alignments and open reading frame (ORF) translation of *LsMT* were carried out with Vector NTI Suite 8.0. BLAST (Basic Local Alignment Search Tool) was performed at the NCBI server (http://www.ncbi.nlm.nih.gov/blast/Blast.cgi). ChloroP1.1 analysis (http://www.cbs.dtu.dk/services/ChloroP/) was done to predict the possible chloroplast transit peptide (cTP) of LsMT.

### Transformation of lettuce by *LsMT*

The coding region of *LsMT* with a *Bam*HⅠ and *Sac*Ⅰ restriction site on either end respectively was cloned into pCAMBIA 2300 expression vector under the control of 35S promoter to generate pCAMBIA 2300-35S:*LsMT*:Noster construct. The construct was transduced into Agrobacterium tunefaciens strain EHA105 and then introduced into lettuce to generate *LsMT*-transgenic plants as previously described [47]. Independent transgenic lines were grown and selected in kanamycin-containing MS medium. *LsMT* transformants were confirmed by PCR detection with primers MT-FP (5'-AGGGAAGGCTTGTTTAATCGGTC-3') and Nos-RP (5'-TAATCATCGCAAGACCGGCAAC-3'). The pCAMBIA 2300-35S:*LsMT*:Noster construct was used as the positive control in PCR analysis. T1 progenies of transgenic lines were kept for further analyses.

### qPCR analyses of *LsMT* transformants

Quantitative real-time PCR (qRT-PCR) was conducted to detect the distinction of expression levels of *HPPD*, *HPT*, *MPBQ-MT*, *TC* and *γ-TMT* between transgenic and non-transgenic plants. Aliquots of 0.6μg total RNA was employed in reverse transcription for cDNA synthesis. The amplification reaction of qRT-PCR was performed according to the recommended protocol of SYBR ExScript RT-PCR Kit (Takara, Japan) on a Real-Time PCR Device (BioRad, Watford, UK). The specific primers for each gene used in qPCR were: HPPD-RT1/2, HPT-RT1/2, MT-RT1/2, TC-RT1/2 and γTMT-RT1/2 respectively (see S1 Table for primers’ sequences). *Ubiquitin* was used as a control for normalization of gene expression. The primers for *Ubiquitin* used in qPCR were Ubi-FP and Ubi-RP (S1 Table). mRNA expression levels of the five genes in transformants and WT were measured with 2^-ΔCt^.

### Analyses of tocopherols, carotenoids, chlorophyll, phylloquinone and ascorbate

For tocopherol and phylloquinone analyses, 0.1g freeze-dried powder derived from leaves of 3-month-old plants was added into 3ml CHCl_3_/n-hexane (3:7, v/v) solvent under dim light and ultrasonically oscillated for 10min. After centrifugation at 5000g for 8min, the clear supernatant was taken and the extraction was repeated twice more. The resulting supernatant was evaporated to dryness under nitrogen and dissolved in 1.5ml CHCl_3_/methanol (3:7, v/v). 30μl of the sample solution was used for reverse phase-HPLC analysis. All the standards of α-, γ-, δ-tocopherol and phylloquinone were purchased from Sigma-Aldrich (Shanghai, China). Target analytes were separated on a YMC Carotenoid C30 Column (250×4.6mm, 5μm) under the following condition: 97% methanol for 6min, 97%-100% methanol in 10min, 100% methanol for 20min, 100%-97% methanol in 2min, and re-equilibration at 97% methanol for 6min at a flow rate of 0.8ml/min. Analytes were detected by photodiode array detector (PDA) at 292nm (S4 Fig). Ascorbate content was measured by means of dichloroindophenol titration, according to the method described in [48]. Total carotenoid and chlorophyll contents were measured with spectrophotometry, according to the method described in [49].

### MRM analysis of PQ level in lettuce

0.5g fresh leaves were homogenized in liquid nitrogen and 2.5ml cold acetone/n-hexane (4:1, v:v) was used for extraction of PQ under dim light. After centrifugation at 7000g for 4min, the supernatant was taken. The extraction was repeated twice more. The total supernatant was evaporated to dryness under nitrogen, dissolved in 1ml ethanol and diluted for 10 times. The resulting sample solution was used for multiple-reaction monitoring (MRM) scan on a UPLC-3Q 5500 system in the positive ion mode. Samples were separated on an Acquity UPLC BEH C18 column (50×2.1mm, 1.7μm) under the following condition: 90%-100% acetonitrile in 1.5min, 100%-90% acetonitrile in 1min, and 90% acetonitrile for 0.5min at a flow rate of 0.8ml/min. The column temperature was set at 40°C. The collision energy was 40V. The nebulizing current was 5.0μA. The entrance potential was 10V, with declustering potential 100V. The source temperature was 500°C. The characterized parent ion of 749.6Da and daughter ion of 151.1Da were specifically extracted for MRM analysis of PQ level.

### Measurements of chlorophyll fluorescence, photooxidation products, thermal dissipation (NPQ) and soluble sugars under high light stress

The maximum quantum yield of PS II photochemistry (Fv/Fm) and the actual quantum yield of PS II-mediated electron transport (ΦPS II) were measured with a fluorescence monitoring system-2 (FMS-2) (Hansatech, UK) according to the instructions of the apparatus. NPQ value was calculated by Fm/Fm’-1, with Fm and Fm’ being maximum fluorescence measured after dark adaption and in light-adapted conditions separately. Malondialdehyde (MDA), the end-product of lipid peroxidation, was detected through reaction with thiobarbituric acid (TBA). H_2_O_2_ level was detected through reaction with TiSO_4_. Total soluble sugars were measured by anthrone colorimetry method. The assays of MDA, H_2_O_2_ and soluble sugars were performed according to the protocols of the assay kits (Solarbio Co., Peking, China) respectively.

## Supporting Information

S1 Fig Full-length sequence of *MPBQ-MT* from lettuce comprising introns and exons. Exons are shown with yellow background while introns are shown with white background.
(DOCX)

S2 Fig Sequence alignment of MPBQ/MSBQ-MTs. Amino acid sequences were aligned as follows: LsMT (*Lactuca sativa*, ACP43457), HaMT1/MT2 (*Helianthus annuus*, ABB52805/ABB52808), AtMT (*Arabidopsis thaliana*, AEE80478), BnMT (*Brassica napus*, ACD03289), DkMT (*Diospyros kaki*, BAL46505), CrMT (*Chlamydomonas reinhardtii*, EDP03731), OsMT (*Oryza sativa*, Q6ZLD3), TaMT (*Triticum aestivum*, CAX36917). The completely identical amino acids were shown with capital letters against black background. Less conserved amino acids were shown with capital letters against dark-grey or relatively light-grey background. Non-conserved amino acids were shown with capital letters against white background. Consensus sequence was shown below the sequences aligned.
(TIF)

S3 Fig PCR detection of T1 progenies of *LsMT* transgenic lines. M: DL 2000 DNA marker; +: positive control; CK: wild type plant; M1-M4: 4 independent transgenic lines, 3–4 T1 progenies of which were selected for PCR detection.
(TIF)

S4 Fig HPLC profiles of the analyzed metabolites: tocopherols (Toc) and vitamin K1 (Vk1). (A). HPLC profiles of standards (STD) of tocopherols and vitamin K1; (B). HPLC profiles of tocopherols and vitamin K1 in wild type samples (WT); (C). HPLC profiles of tocopherols and vitamin K1 in transgenic samples (TR).
(TIF)

S5 Fig A schematic image indicating the process of photosynthetic electron transport and photoprotection. Abbreviations: AsA, ascorbate; Fd, ferredoxin; FNR: Fd-NADP⁺ reductase; FQR, Fd-plastoquinone oxidoreductase; LHC, light-harvesting complex; MDA, monodehydroascorbate radical; NDH, NADPH/NADH dehydrogenase; NPQ, non-photochemical quenching; OEC, oxygen-evolving complex; PC, plastocyanin; SOD, superoxide dismutase; VDE, violaxanthin de-epoxidase; vio, violaxanthin; zea, zeaxanthin.
(TIF)

S1 Table Gene specific primers used for qRT-PCR analysis. (DOCX)
