# Supplementary material for: Roles of MPBQ-MT in Promoting α/γ-Tocopherol Production and Photosynthesis under High Light in Lettuce
Source: PLoS One. 2016 Feb 11;11(2):e0148490. doi: 10.1371/journal.pone.0148490 (PMC4750918; doi:10.1371/journal.pone.0148490)
Supplement: S1 Fig — Exons are shown with yellow background while introns are shown with white background. (DOCX) [file pone.0148490.s001.docx]

ATGGCTTCGTCGATGCTCTATGGAGGTCAGCATCTTACATTAACCCCAAAAGGGTTAGGGTTTAATGGGTCAGATTTGCATGGTAAGAATTTGCTGAAGATGAATTTGGTTGGTAGAAATTGTAATTTTCGATCTAAAACGCTAGTTGTACCCAAATGCAGCGTGTCTGTACCAAGGCCAGCTTCACAGCCTAGGTTCATACAGCATAAAAAAGAGGCATTTTGGTTTTATAGGTTTCTGTCAATCGTTTACGATCATGTGATAAACCCTGGTCATTGGACTGAAGATATGCGAGACGATGCACTCGAACCAGCTGATCTTAATAGCCGCGATTCAATAGTGGTGGATGTTGGTGGAGGCACTGGGTTCACTACTTTAGGTATTGTAAAACATGTCGATGCTAAAAATGTTACCATTTTGGATCAATCGCCTCATCAGCTTGCAAAAGCTAAAGAGAAGGAGCCTTTGAAGGAATGCAAGATCATTGAAGGAGATGCTGAAGATCTTCCTTTCAAAACTGATTATGCCGATAGATATGTGTCAGCCGGAAGGTATAGCCTCTAATAAATGTTCATTTCTATGTGATTACTGCTATTTGTGTAATCATTAGATAATCATATAATTAATTGCTTGAGGGGGTGTTTAGATGTATGATTAAACCTATGATCGTGACAATCGCGTGTCTCTGTAAACTGATTATATGATTTCTGAAATGGTGAAAACAATAAGTTATGAATAGTCAATGAAAGAATTGGCAAAAGAAAGATGATTATTGGGATACATCTAAATGCCCAATTATCCATAGCTTTAAGCAACTGATTATCTTATTCCTCAAATGGTGAAAGAATTGCCATATGTCCAGAAAGATGTATGTTTAGTGTCCAATTATATATCCTTTGGAAACATATGAGTTGAAAGTGGGTAGATGTAAGTAACACCCCTGTCCAATCCAAACACTGTGCAATTTGTTATGTCATGTCATCCCAGTCCAATTGAAATACATATGTCATGTCATCCCAGTCCAATTGAAATACAAAATACAGTACAACCTGTTATGTCATGTCATGTATTCTGACCATATTTCTTTTCAGCATTGAGTACTGGCCGGACCCACAACGAGGCATCAAAGAAGCATACAGGGTTTTGAAGATGGGAGGGAAGGCTTGTTTAATCGGTCCTGTTTATCCGACATTTTGGTTGTCTCGTTTCTTTGCAGACATGTGGATGCTCTTTCCTAAAGAGGAAGAGTACATGGAGTGGTTTGAAAAGGCAGGATTCAAAGATGTTGAGATTAAAAGGATTGGCCCAAAATGGTATCGCGGGGTCCGCCGACATGGTCTCATCATGGGATGCTCTGTGACAGGTGTCAAACCCACGTCAGGGGACTCTCCTTTGCAGGTAACCTCTTCTTCCTCATTACCAATAATGATGATATATATTGATATGGTTAAAAAGTGAATAATAATTTAAAAAGGGTTTTTTTTTGTAATTTTTGTTGCAGCTTGGTCCAAAGGTAGAGGACATAGAGACGCCTGTAAATCCATTTGTGTTCCTTTTACGTTTCCTTCTTGGGGCATCAGCTGGAGTGTACTATGTTTTGGTTCCTGTTTACATGTGGATCAAGGACCAGATTGTGCCAAAAGGTCAACCAATTTGA
