# Supplementary figures and images for: Roles of MPBQ-MT in Promoting α/γ-Tocopherol Production and Photosynthesis under High Light in Lettuce
Source: PLoS One. 2016 Feb 11;11(2):e0148490. doi: 10.1371/journal.pone.0148490 (PMC4750918; doi:10.1371/journal.pone.0148490)

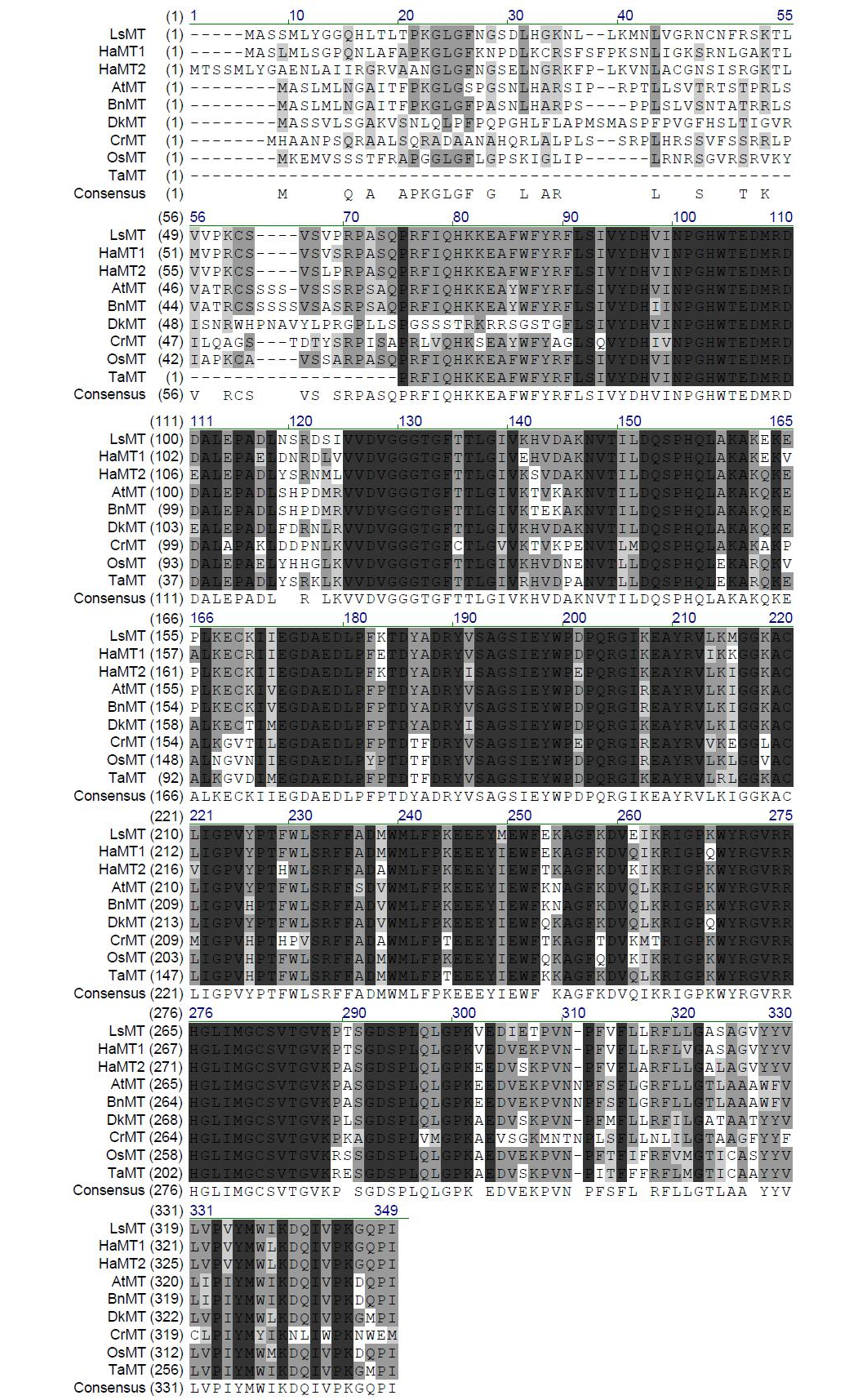

Supplement: S2 Fig — Amino acid sequences were aligned as follows: LsMT (Lactuca sativa, ACP43457), HaMT1/MT2 (Helianthus annuus, ABB52805/ABB52808), AtMT (Arabidopsis thaliana, AEE80478), BnMT (Brassica napus, ACD03289), DkMT (Diospyros kaki, BAL46505), CrMT (Chlamydomonas reinhardtii, EDP03731), OsMT (Oryza sativa, Q6ZLD3), TaMT (Triticum aestivum, CAX36917). The completely identical amino acids were shown with capital letters against black background. Less conserved amino acids were shown with capital letters against dark-grey or relatively light-grey background. Non-conserved amino acids were shown with capital letters against white background. Consensus sequence was shown below the sequences aligned. (TIF) [file pone.0148490.s002.tif]

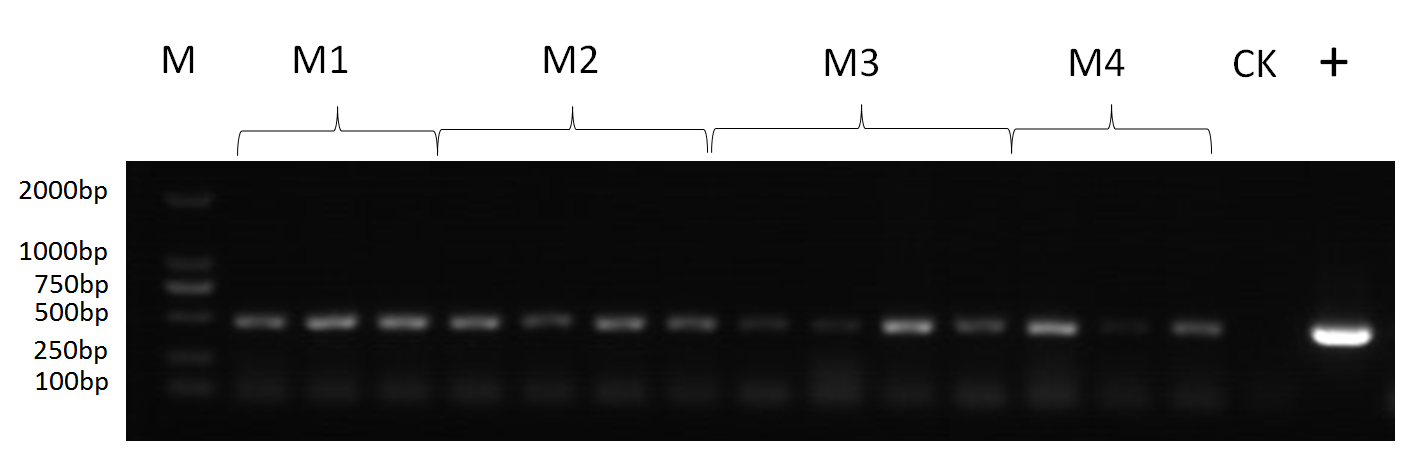

Supplement: S3 Fig — M: DL 2000 DNA marker; +: positive control; CK: wild type plant; M1-M4: 4 independent transgenic lines, 3–4 T1 progenies of which were selected for PCR detection. (TIF) [file pone.0148490.s003.tif]

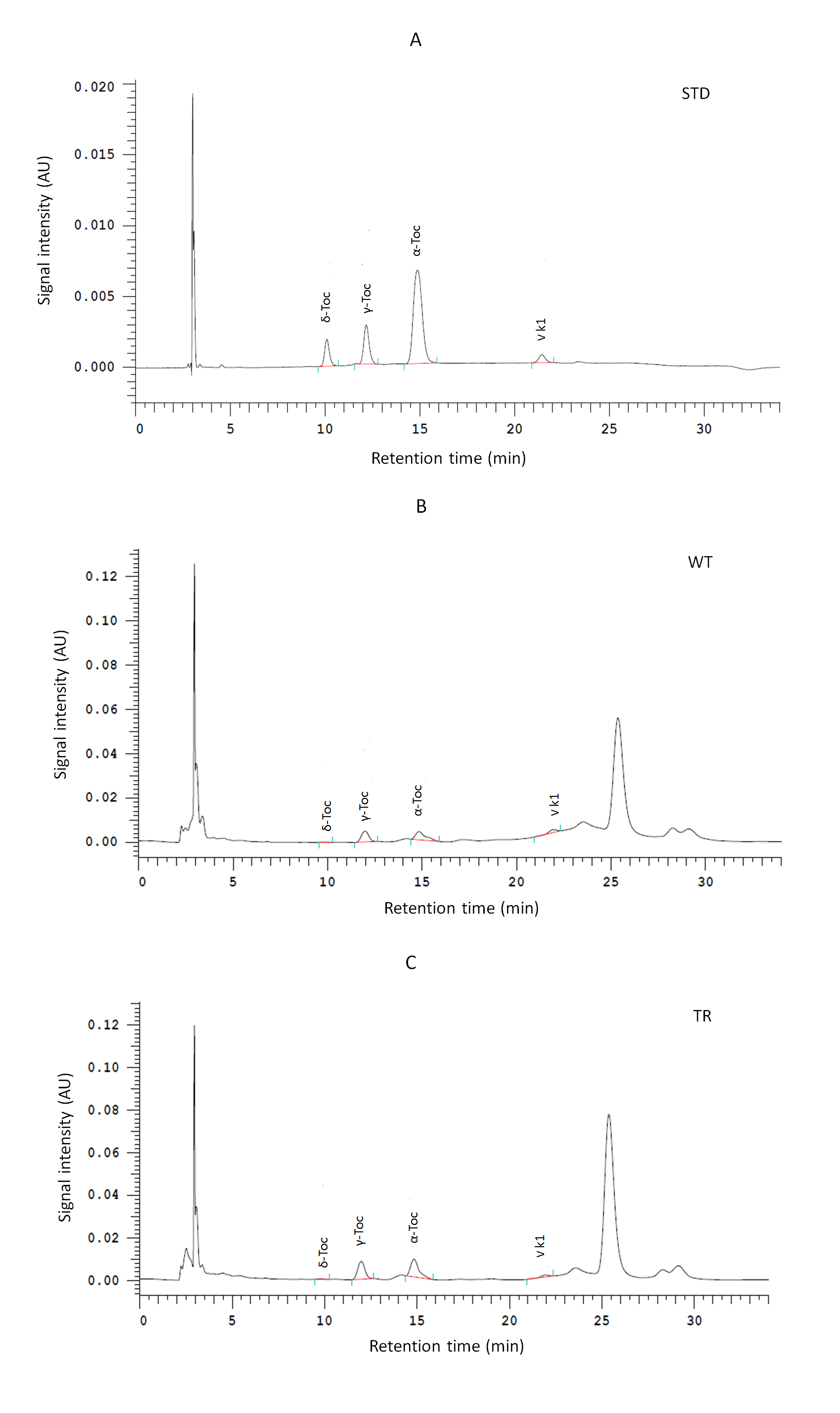

Supplement: S4 Fig — (A). HPLC profiles of standards (STD) of tocopherols and vitamin K1; (B). HPLC profiles of tocopherols and vitamin K1 in wild type samples (WT); (C). HPLC profiles of tocopherols and vitamin K1 in transgenic samples (TR). (TIF) [file pone.0148490.s004.tif]

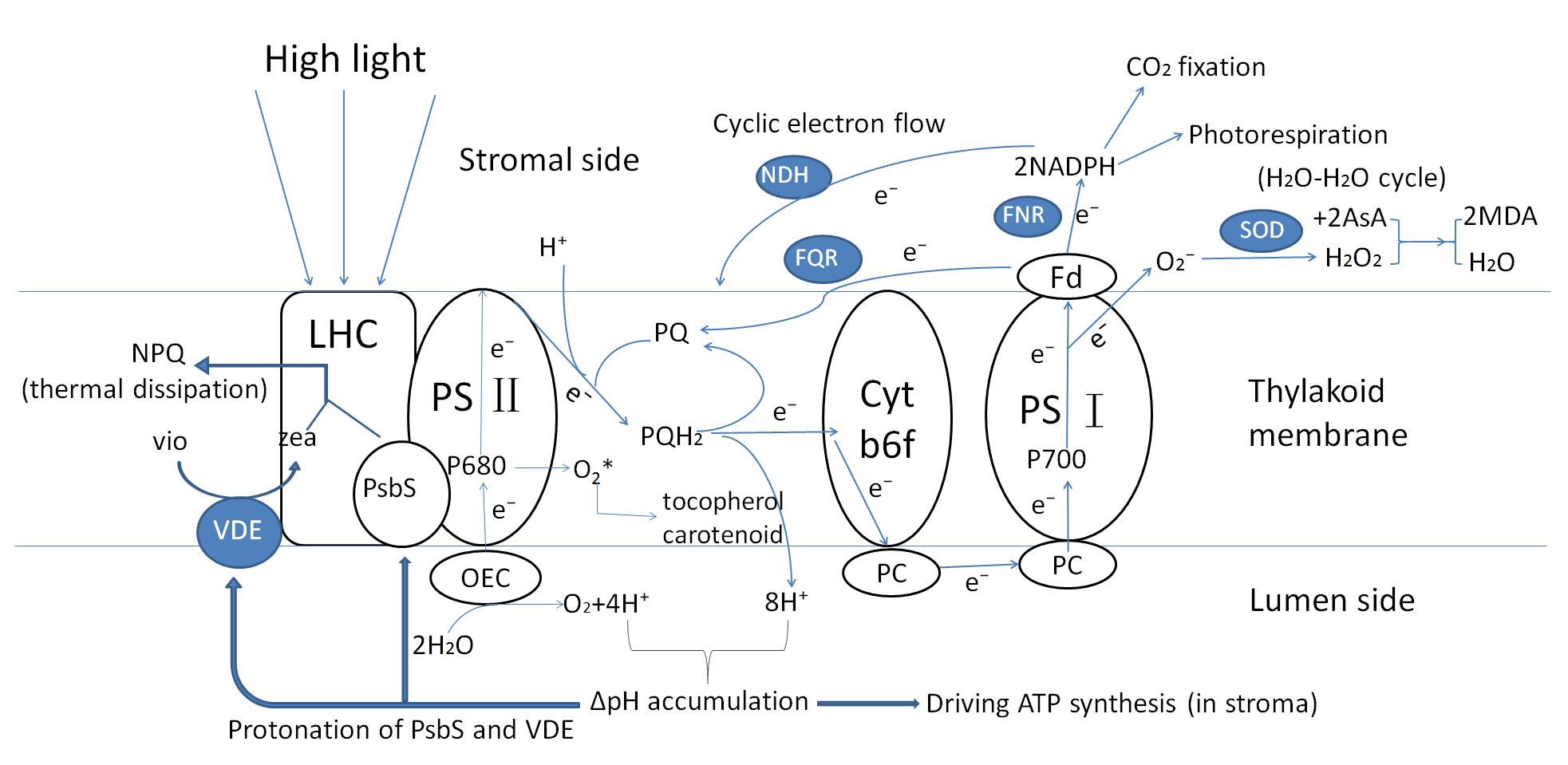

Supplement: S5 Fig — Abbreviations: AsA, ascorbate; Fd, ferredoxin; FNR: Fd-NADP⁺ reductase; FQR, Fd-plastoquinone oxidoreductase; LHC, light-harvesting complex; MDA, monodehydroascorbate radical; NDH, NADPH/NADH dehydrogenase; NPQ, non-photochemical quenching; OEC, oxygen-evolving complex; PC, plastocyanin; SOD, superoxide dismutase; VDE, violaxanthin de-epoxidase; vio, violaxanthin; zea, zeaxanthin. (TIF) [file pone.0148490.s005.tif]
