# Supplementary material for: Roles of MPBQ-MT in Promoting α/γ-Tocopherol Production and Photosynthesis under High Light in Lettuce
Source: PLoS One. 2016 Feb 11;11(2):e0148490. doi: 10.1371/journal.pone.0148490 (PMC4750918; doi:10.1371/journal.pone.0148490)
Supplement: S1 Table — (DOCX) [file pone.0148490.s006.docx]

| Primer name | Sequence (5’ → 3’) |
| --- | --- |
| HPPD-RT1 | TGTATGGTGAAAGATGGTGAAGGCAAGG |
| HPPD-RT2 | CAGCAGCAGTTGGTTCAGTAGTGGTTCG |
| HPT-RT1 | TATTACTTGAAGTGGCTTATGG |
| HPT-RT2 | ATCAGTTGACTTAGCACGACCC |
| MT-RT1 | TCACAGCCTAGGTTCATACAGCA |
| MT-RT2 | CAGTCCAATGACCAGGGTTTATC |
| TC-RT1 | AAATCGACCTCTTCGTACTCCC |
| TC-RT2 | TCCATACTGTAATTGCTCCAAAC |
| γTMT-RT1 | CATCACCCTCAGCCCTGTTC |
| γTMT-RT2 | TCCGCAACTTGAAATGAAACC |
| Ubi-FP | AAGACCTACACCAAGCCCAA |
| Ubi-RP | AAGTGAGCCCACACTTACCA |
